# CTLA-4 (+49A/G) Polymorphism in Type 1 Diabetes Children of Sudanese Population

**DOI:** 10.1055/s-0041-1723008

**Published:** 2021-02-15

**Authors:** Khalid E. Khalid Kheiralla

**Affiliations:** 1 Department of Basic Medical Sciences, Faculty of Applied Medical Sciences, Albaha University, Al Bahah, Saudi Arabia; 2 Department of Biochemistry and Nutrition, Faculty of Medicine, University of Gezira, Wad Madani, Sudan

**Keywords:** CTLA-4, polymorphism, type 1 diabetes Mellitus, Sudanese

## Abstract

**Background** Type 1 diabetes mellitus (T1DM) is an organ-specific T cell-mediated autoimmune disease, characterized by destruction of pancreatic islets. Cytotoxic lymphocyte antigen-4 ( *CTLA-4* ) is a negative regulator of T cell proliferation, thus conferring susceptibility to autoimmunity.

**Aims** This study aimed to investigate the association of *CTLA-4* +49A/G (rs231775) polymorphism with a risk of T1DM in Sudanese children.

**Methods** This a case–control study included 100 children with T1DM, referred to the pediatric clinic at referral pediatric teaching hospital in Gezira State-Sudan. Hundred unrelated healthy controls were recruited from departments in the same hospital. Genomic deoxyribonucleic acid (DNA) was extracted from Ethylenediaminetetraacetic Acid (EDTA)-preserved blood using QIAamp DNA Blood Mini Kit (QIAamp Blood) (QIAGEN; Valencia, CA). The polymerase chain reaction PCR restriction fragment length polymorphism (PCR-RFLP) and sequencing were applied for the *CTLA-4* (+49A/G) genotyping. The changes accompanied the polymorphism were evaluated using relevant bioinformatics tools.

**Results** The genotype and allele frequencies of the *CTLA-4* (+49A/G) polymorphism were significantly different between the patients and controls ( *p* = 0.00013 and 0.0002, respectively). In particular, the frequency of the G allele, GG homozygous genotype, and AG heterozygous genotype were significantly increased in patients than in controls ([28% versus 7%, odds ratio (OR) = 5.16, 95% confidence interval [CI] = 2.77–9.65, *p* = 0.00] [12% versus 2%, OR = 6.68, CI = 1.46–30.69, *p* = 0.01] [32% versus 10%, OR = 4.24, CI = 1.95–9.21, *p* = 0.00], respectively). The presence of the G allele (homozygous) showed an influence on the signal peptide polarity, hydrophobicity, and α-helix propensity of the CTLA-protein.

**Conclusion** The results further support the association of *CTLA-4* (+49A/G) polymorphism and the risk of T1DM in our study population.

## Introduction

Type 1 diabetes mellitus (T1DM) is an organ-specific and T cell-mediated autoimmune disease primarily affects children and adolescents. Cytotoxic lymphocyte antigen-4 ( *CTLA-4* ) is a member of the immunoglobulin superfamily that is expressed on the surface of activated T cells and downregulates T cell function. 1 In the mid-nineties, the *CTLA-4* gene was reported as one of the important susceptibility genes in T1DM, 2 since that time, attentions have been made toward the exact role of this gene. Polymorphisms in the *CTLA-4* gene have been identified and were found to be associated with susceptibilities to a wide range of T cell-mediated autoimmune diseases. 3

One of these polymorphisms was the *CTLA-4* +49A/G single nucleotide polymorphism (SNP) that causes a threonine-to-alanine substitution in codon 17, which altered protein expression 4 and T cell activation. 5 Since the +49A/G SNP is located in the N-terminal of the signal peptide sequence of the *CTLA-4* , which is not a part of the mature protein, the substitution of threonine to alanine may affect the proper translocation of the growing *CTLA-4* peptide from ribosome to endoplasmic reticulum (ER) lumen, as a result of alteration in signal peptide hydrophobicity and helix propensity, 6 rendered possible evidence of defective CTLA-4 targeting to the cell surface. 7

The association of CTLA-4 polymorphisms with the risk to develop T1DM has been investigated in different populations with conflicting data. 8 Recent study has shown no association between the aforementioned SNPs and susceptibility toT1DM among Sudanese adults. 9 Since Sudanese population is characterized by multiethnic groups, the search for further association between groups of different age and ethnicity could likely help pursuing conclusive remarks about this association. In the present study, we investigated the association of the *CTLA-4* +49A/G SNP with the risk of T1DM among Sudanese children and the proposed effect of this polymorphism on the *CTLA-4* signal peptide instability.

## Subjects and Methods

### Patients and Sampling

This is a case–control study that encompasses 100 Sudanese children with T1DM (48 males and 52 females; mean age, 11.49 ± 3.38), referred to the diabetic clinic at Wad Medani Pediatric Hospital in Gezira state, Sudan. The selected patients were clinically diagnosed with T1DM, their disease duration was more than 1 year, and they are dependent on insulin therapy. The patients were classified based on the hemoglobin A1c (HbA _1c_ ) levels into poor glycemic control >8% and well glycemic control ≤8%, as stated on the American Diabetes Association (ADA) and Japan Diabetes Society (JDS) guidelines. 10 11 The demographic characteristics, clinical presentations, Hb _A1c_ levels, concomitant complications, and the presence of other autoimmune diseases were all reported in well-structured questionnaire. The control group includes 100 unrelated healthy children (44 males and 56 females; mean age, 11.49 ± 3.38) without or family history of T1DM or any other autoimmune diseases. The controls were recruited from departments in the same hospital, they lived in the same state, and they have ethnic background similar to the patients.

The study met the University of Gezira ethical committee review board requirements, and granted the permission to be performed from the hospital clinical directorate and the diabetic clinical staff as well. Signed written informed consent was obtained from the parents or guardians of all study subjects, after they informed about the study objectives and procedures.

Five-milliliter (5 mL) blood sample was taken in the morning (before breakfast) in EDTA container. Serum was separated to measure the HbA _1c_ by chromatographic-spectrophotometer ion exchange (BioSystems, United States), and the pellet used for the deoxyribonucleic acid (DNA) analysis of *CTLA-4* +49A/G genotypes.

### DNA Extraction and CTLA-4 Amplification

Genomic DNA was extracted using QIAamp DNA Blood Mini Kit (QIAamp Blood) (QIAGEN, Valencia, CA). The desired fragment of the *CTLA-4* gene was amplified by polymerase chain reaction (PCR) using *CTLA-4* gene-specific forward (5̀-gCTCTACCTCTTgAAgACCT-3̀) and the reverse (5̀-AgTCTCACTCACCTTTgCAg-3) primers, which amplified 207 fragments of *CTLA-4* gene.

**Fig. 1:**
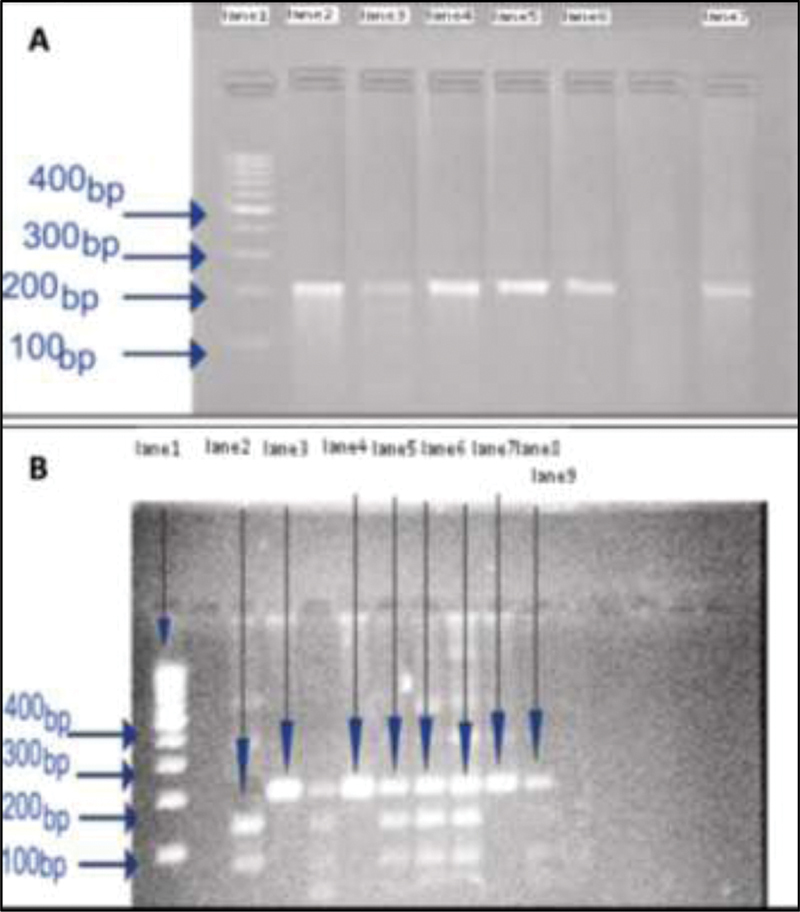
( **A** ) Confirmation of the genomic deoxyribonucleic acid (DNA) in patients and controls. Lane 1: DNA ladder 100 bp. The band appeared in lane 2 to 7 showed the polymerase chain reaction (PCR) product length of 207 bp for the CTLA-4 target gene in exon 1. ( **B** ) Fragment size for CTLA-4 (+49A/G) polymorphism in diabetic patients and controls by FastDigest BbvI (LSP1109I, Germany). Lane 1: DNA ladder 100 bp; the bands in lane 3, 4, 7, and 8 represent allele A at 207 bp, whereas fragments of band G at 168 and 49 bp appear in lane 2, 5, and 6. Lanes 5, 6, 7: heterozygous (AG) genotype. Lanes 3, 4, 7, 8: homozygous (AA) genotype. Lane 2 homozygous (GG). CTLA-4, cytotoxic T-lymphocyte antigen-4.

Approximately, 0.2 mg genomic DNA was amplified in 25 mL PCR reaction containing 10 mM of each dNTPs (i-StarTaq, iNtRon Biotech, Korea), 5 U of Taq DNA polymerase (i-StarTaq, Korea), 2.5 mL of 10X PCR buffer, and 100 pmol/mL of each primer (Sinagen, Iran). Reaction conditions were performed in PCR thermocycler (Eppendorf, Germany), starting with initial denaturation at 94°C for 4 minutes followed by 34 cycles of denaturation at 94°C for 30 second annealing at 60°C for 30 seconds elongation at 72°C for 2 minutes, and final extension at 72°C for 5 minutes. Then 5 μL of the PCR product was run in 2% agarose gel electrophoresis to check the target PCR product at 207 bp length ( Fig. 1A ).

### Restriction Fragment Length Polymorphism Analysis

Restriction fragment length polymorphism (RFLP) analysis was conducted using FastDigest BbvI (Fermentas, Germany) in 30 μL total volume that includes 10 μL amplification product, 1.0 μL (10 U/mL) of the restriction enzyme, 2.0 μL 10X fast digest green buffer, and 17 μL nuclease-free water. All components were mixed gently, spinned down, and incubated for 10 minutes at 37°C followed by heating at 65°C for 10 minutes. DNA fragments were visualized in 2.0% agarose gels exposed to UV light in a gel documentation system (Ingenus, United States). The restriction cut showed two fragments (49/168 bp) for the G allele and one fragment (207bp) for A allele ( Fig. 1B ). Direct DNA sequencing by Sanger method on an ABI 3730 sequencer (Macrogen, South Korea) was performed for 20 patients and 20 control by using 25 μL of each PCR result independently, to validate the RFLP results. The sequencing results were analyzed using BLAST and Clustal X at the NCBI webpage.

### Bioinformatics Analysis Tools

I-mutant version 3 ( http://gpcr2.biocomp.unibo.it/cgi/predictors/1-Mutant3.0/ ) was used to predict the effect of the *CTLA-4* +49A/G SNP in proteins stability. 12

Sorting Intolerant from Tolerant (SIFT) was used to predict whether an amino acid substitution affects protein function or not, based on the degree of amino acids conservation residues in sequence alignments derived from closely related sequences. The main underlying principle of this program is that it generates alignments with large number of homologous sequences, and assigns scores to each residue ranging from zero to one. Score close to zero indicates evolutionary conservation of the gene and intolerance to substitution, while score close to one indicates only tolerance to substitution. 13 The algorithmic calculation methods of the hydrophobicity 14 and α-helix propensity 15 that accompanied the *CTLA-4* T17A change were also determined to help in predicting the alteration in the *CTLA-4* signal peptide function.

### Data Analysis

Statistical analysis was performed using SPSS software package, version 23 (SPSS, Inc.; Chicago, Illinois, United States). The mean difference between study group was assessed by using the Student's *t* -test. Qualitative data were presented as number of (%), the comparisons of genotypes and alleles frequencies between patients and controls were assessed using the chi-squared (x ^2^ ) test and the Fisher's exact tests, and levels of risk for genotypes and alleles were expressed as odds ratio (OR) with a 95% confidence interval (95% CI). Deviation from Hardy–Weinberg equilibrium was performed by applying the equation (p 2 + 2pq + q 2 ) to compare the observed frequencies with the expected frequencies of the different genotype distribution in patients and controls by using Pearson's x ^2^ test of independence in SPSS. Statistical significance was considered at *p* <0.05.

## Results

The study included 100 children with T1D (48 males and 52 females) and 100 unrelated healthy controls (44 males and 56 females).

Table 1 showed the sociodemographic and clinical characteristics of study groups. There were no significant differences in the age and gender between patients and controls ( *p* > 0.05). Compared with the controls, the patients showed significant high mean levels of HbA _1c_ ( *p* = 0.0003) and low mean level of body mass index ( *p* = 0.01).

**Table 1:** Demographic characteristics of the patients and the controls

Characteristics		T1DM ( *n* = 100)	Controls ( *n* = 100)
Gender	Male	48	56
	Female	52	44
Age group/yrs.	Mean ± SD	11.49 ± 3.38	10.24 ± 4.31
	<5	2	24
	5–9.9	26	38
	10–14.9	50	30
	≥15	22	8
Residence	Rural	80	54
	Urban	2 (2.0)	46
Insulin dependent		All	None
Age at disease onset (years)		3–16	NA
T1DM duration		4.35 ± 3.02	NA
Family history of diabetes	Negative	64	NA
	Positive	36	NA
Glycemic control	HbA _1c_ mean level	10.68 ± 2.17	6.05 ± 1.40 a
	HbA _1c_ <8%	12	94
	HbA _1c_ >8%	88	6
Breast feeding	Yes	45	NA
	No	55	NA
BMI (kg/m ^2^ )	Range	16.74 ± 2.90	19.4 ± 4.74 a
	Underweight (<18.5 kg/m ^2^ )	80	52
	Normal (18.5–24.9 kg/m ^2^ )	18	28
	Overweight (25–29.9 kg/m ^2^ )	1	16
	Obese (≥30 kg/m ^2^ )	1	4
On treatment	Regular	All	NA
	Not regular	−	NA
Disease complications	Eye complication	9	NA
	Renal complication	1	NA
	Hypoglycemia	45	NA
	Ketoacidosis	63	NA
Autoimmune diseases	No	92	NA
	Celiac disease	8	NA

Abbreviations: BMI, body mass index; HbA1c, hemoglobin A1c; NA, not applicable; SD, standard deviation; T1DM, type 1 diabetes mellitus.

a *p* < 0.05.

As shown in Table 2 , the frequency distribution of CTLA-4 +49 A/G genotypes and alleles showed significant differences between the patients and the controls ( *p* = 0.00013 and 0.0002, respectively). The genotypic distribution was not deviated from the Hardy-Weinberg equilibrium in patients ( *x* ^2^ = 5.19, df = 2, *p* = 0.08) and controls ( *x* ^2^ = 4.82, df = 2, *p* = 0.09).

**Table 2:** Genotypes and alleles frequencies of CTLA-4 +49A/G polymorphism in T1DM patients and controls

CTLA-4 variants	T1DM ( *n* = 100)	Controls ( *n* = 100)	OR	95% CI	*p* -Value
** Genotype frequencies a**					
AA (normal)	56 (56)	88 (88)	0.17	0.08–0.36	0.00
AG (heterozygous)	32 (32)	10 (10)	4.24	1.95–9.21	0.00
GG (homozygous)	12 (12)	2 (2)	6.68	1.46–30.69	0.01
** Allele frequencies b**					
* A allele	144 (72)	186 (93)	1.94	0.10–0.36	0.00
** G allele	56 (28)	14 (7)	5.16	2.77–9.65	0.00

Abbreviations: CI, confidence interval; CTLA-4, cytotoxic T-lymphocyte antigen-4; OR, odds ratio; T1DM, type 1 diabetes mellitus.

a *p* = 0.00013.

b *p* = 0.0002.

* Adenine.

** Guanine.

The GG homozygous and AG heterozygous genotypes were more frequent in patients than in controls (12 vs. 2% and 32 vs. 10%, respectively). This difference was statistically significant ( *p* = 0.01, OR = 6.68, 95% CI = 1.46–30.69 vs. *p* = 0.00, OR = 4.24, 95% CI = 1.95–9.21, respectively). At the same time, the *CTLA* Ala 16 (G) allele was significantly high in frequency in patients than in controls (28 vs. 7%, OR = 5.16, 95% CI = 2.77–9.56, *p* = 0.00).

**Fig. 2:**
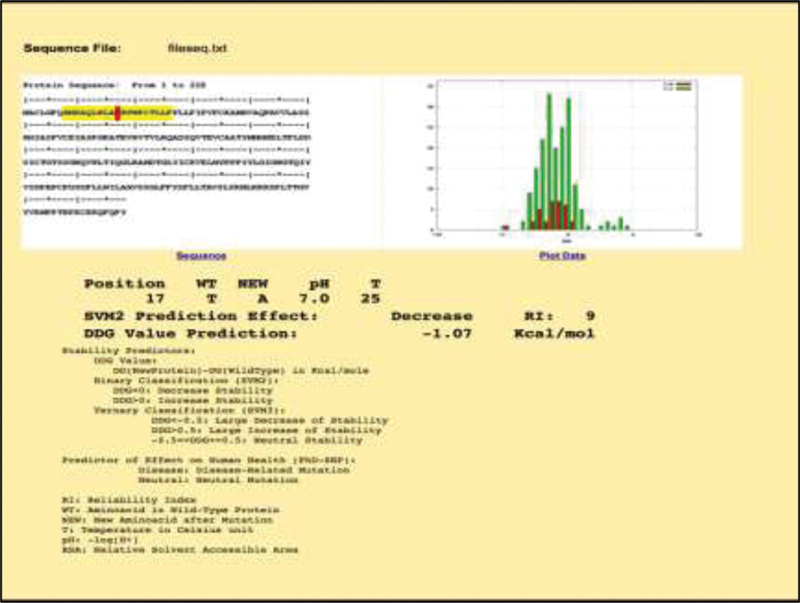
Prediction of protein stability by 1-mutant program.

**Fig. 3:**
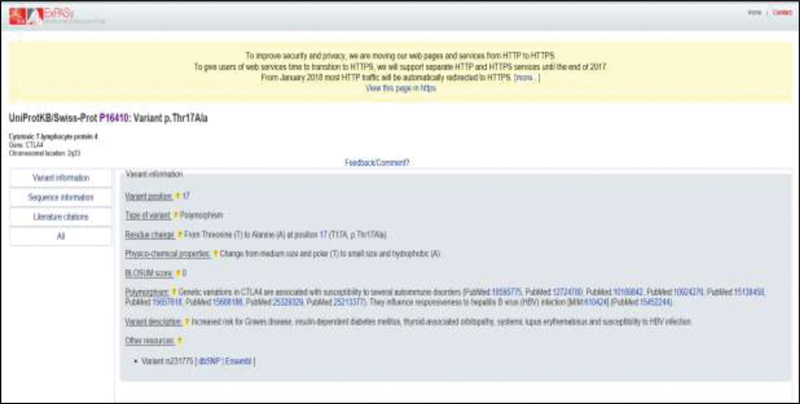
The variation formation of the CTLA-4 SNP. This figure shows the variant formation for the CTLA-4 (rs231775) SNP position at codon 17 (T/A), this SNP affects the polarity (from polar threonine to nonpolar alanine) of the CTLA-4 signal peptide chain. CTLA-4, cytotoxic T-lymphocyte antigen-4; SNP, single nucleotide polymorphism.

**Fig. 4:**
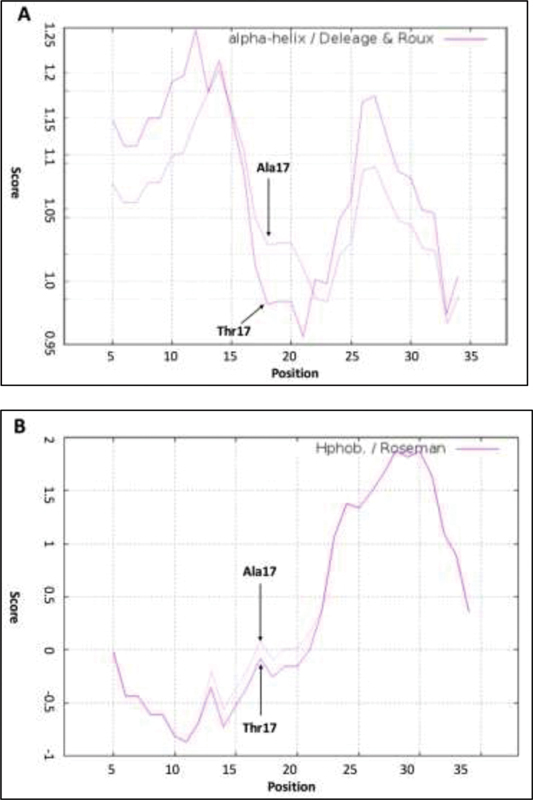
The prediction of signal peptide function. Signal peptide function could be influences by factors that can be predicted by using algorithms available at ProtScale (us.expasy.org/cgi-bin/protscale.pl). ( **A** ) α-helix propensity was calculated by Roseman method (1988), and ( **B** ) hydrophobicity by the Deléage and Roux Method (1987). The CTLA-4 T17A change resulted in a higher propensity to form α-helices in the area directly adjacent to the change and an increased in hydrophobicity. CTLA-4, cytotoxic T-lymphocyte antigen-4.

SIFT analysis score for the *CTLA-4* SNP (rs231775) position at codon 17 (T17A) indicates evolutionary conservation of the gene and intolerance to substitution ( Table 3 ) that my decrease the *CTLA-4* protein stability as predicted by the I mutant analysis ( Fig. 2 ), most likely by affecting the polarity (from polar threonine to nonpolar alanine) of the *CTLA-4* signal peptide chain ( Fig. 3 ). The threonine to alanine substitution at position 17 also leads to increase in the hydrophobicity and α-helix propensity, two properties known to be important in the *CTLA-4* signal peptide function ( Fig. 4 ).

**Table 3:** The SIFT score for the CTLA-4 SNP (rs231775)

SNP ID	Organism/Build	Ref allele	Alt allele	Amino acid change	Gene name	SIFT score	SIFT prediction
rs231775	Homo_sapiens/GRCh37.74	A	G	T17A	CTLA-4	0.06	Tolerated

Abbreviations: CTLA-4, cytotoxic T-lymphocyte antigen-4; SIFT, Sorting Intolerant from Tolerant; SNP, single nucleotide polymorphism.

## Discussion

T1DM is one of the most frequent chronic diseases in children, and has become health problem in developing countries. 17 Recently, associated studies have been conducted to address the association of polymorphisms in the CTLA-4 gene as a candidate gene with several autoimmune diseases, 6 particularly T1DM. 7 The CTLA-4 +49A/G gene polymorphism was found to be the only known SNP that causes an amino acid exchange (threonine to alanine or +49 A/G) in exon 1 in the leader peptide sequence of the CTLA-4 protein, which can be associated with altered protein expression 4 and T cell activation. 5

We studied the CTLA-4 (+49 A/G) polymorphism because it has been the most widely analyzed variant in different ethnic groups, and still with inconsistent findings. 16 18 19 20 21 22 23 24 25 26 27 28 29 30 31 32 33 34 35 36 37 38 39 40 41 Furthermore, this is the first study investigated the effect of +49A/G polymorphism among Sudanese children with T1DM, bearing in mind a recent data investigated this polymorphism among Sudanese adults with T1DM. 9

Our study found that the frequency of G allele and GG homozygous genotype was significantly high in patients than in controls ( *p* = 0.01). This finding consistent with previous studies in populations from our continent includes Egyptians 16 18 and Tunisians, 19 together with other populations such as Indian, 20 Caucasian and Asian, 21 22 Iranians, 23 24 25 Estonian and Finnish, 26 Chilean, 27 and Russian. 28 Other studies among Sudanese, 9 Ghanaian, 29 and other populations, 30 31 did not prove this association. The OR provided by the GG homozygous genotype and G allele were high, suggesting their additive effect on the AG heterozygous genotype ( *p* = 0.00) as seen in our study.

The presence of the +49A/G at-risk (G) allele in the CTLA-4 molecule has been shown to be effective in inhibiting the activated T cell proliferation in vitro. 32 This perception coincides with the high frequency of G allele among our patients, and found to be consistent with results from other populations including Egyptians, 16 33 Iranian, 25 Turkish, 34 Croatians, 35 Belgian, 36 Mexican-American, and Korean, 37 and meta-analysis study in Asian population. 38

However, lack of association between the aforementioned polymorphism and T1DM has been also reported in populations from Sudan, 9 Czech, 31 Turkey, 34 Korea, 38 Chile, 39 Portugal, 40 and Azerbaijan. 41 The discrepancy in results between ethnic groups may be attributed to genetic heterogeneity relevant to ethnic diversity, to polymorphism co-players (environmental factors, etc.), or to differences in methodologies and sample size used.

The CTLAAla 16 (G) allele (homozygotes) located at the N-terminal of conserved position in the loop region of the signal peptide sequence introduces the hydrophobic amino acid alanine instead of threonine in the signal peptide sequence. This introduction, and based on our bioinformatics analysis, was somewhat associated with evolutionary conservation of the gene and intolerance that may decrease the CTLA-4 protein stability, affecting the polarity, and increase hydrophobicity and α-helix propensity. These properties are collectively known to be important in signal peptide function. The consequences of these alterations may result in an aberrantly glycosylated product, alteration in proteins folding, and/or interaction with ER chaperones, which may finally lead to less functional expression of CTLA-4 at the cell surface of their T cells than the normal Thr 16 allele. 6 It is most likely that the one-third less expression of the mutant homozygous (GG) on the cell surface of T cells than the normal homozygous (AA) can lower the affinity of CTLA-4 for B7 molecule, skewing the negative balance exerted for damping T cell activation. 7 42

The small sample size in this study, concomitant with the large discrepancy in Sudanese ethnic groups, makes the power of association between the CTLA-4 +49 A/G (rs231775) polymorphism and T1DM relatively weak and the overall data are not fully conclusive.

## Conclusion

The study supported the proposition that CTLA-4 +49 A/G polymorphism is associated with the risk of T1DM in Sudanese children, and the presence of the CTLA-4 Thr 16 (G) allele (homozygous) represents an evolutionary change predisposed the risk for T1DM. This data warrant further studies with larger study population to verify our findings.
